# Prevalence and genotype distribution of HPV infection among women in Xiamen, China

**DOI:** 10.3389/fmicb.2023.1130226

**Published:** 2023-05-30

**Authors:** Ye Shen, Yanru Huang, Wenbo Wang, Jian Zhang, Xiaolu Chen, Lutan Zhang, Xiaomei Huang, Yunsheng Ge

**Affiliations:** Department of Central Laboratory, Women and Children’s Hospital, School of Medicine, Xiamen University, Xiamen, Fujian, China

**Keywords:** human papillomavirus, prevalence, genotype, cervical cancer, infection

## Abstract

**Objective:**

This study aimed to evaluate the prevalence of HPV and genotype distribution among female populations in Xiamen, Fujian Province, China, which can be conducive for local governments to formulate cervical cancer screening and HPV vaccine strategies.

**Methods:**

Cervical swabs were collected from 47,926 participants aged 16–92 years at the Women and Children’s Hospital, Xiamen University, from November 2019 to June 2020. HPV DNA was extracted and detected using conventional PCR, followed by HPV subtype-specific hybridisation. HPV infection rates based on different groups were compared using the *χ*^2^ test. HPV prevalence and the corresponding 95% confidence intervals (95% CI) were calculated using SPSS 19.0.

**Results:**

The overall HPV prevalence among the 47,926 cervical swabs that were analysed was 15.13%, of which single, double, and multiple infections accounted for 76.83, 16.70 and 6.47%, respectively. The age-specific prevalence of HPV infection presented a “U” curve with a HPV prevalence peak observed in women aged <20 years. The gynaecology clinic group had significantly higher HPV positive rates than the health examination group (*p* < 0.001). The five most common HR-HPV subtypes in Xiamen were HPV52, 58, 16, 51, and 39 (2.69, 1.63, 1.23, 1.05, and 0.98%, respectively). The five most common LR-HPV subtypes were HPV54, 61, 81, 70, 34, and 84 (0.92, 0.86, 0.71, 0.45 and 0.35%, respectively).

**Conclusion:**

Our findings demonstrate that the 9-valent HPV vaccine is recommended for regular immunisation in Xiamen. It is necessary for elderly women to participate in HPV screening to decrease the morbidity and mortality of cervical cancer.

## 1. Introduction

Cervical cancer is the fourth most common malignant tumour and the fourth leading cause of death in females threating their health worldwide. Globally, more than 600,000 new cases of cervical cancer are newly diagnosed annually, and the disease resulted in over 34,000 deaths in 2020 (Bruni et al., 2022). Of the 311,365 women who died from the disease, 90% lived in low-and middle-income countries (LMICs) (Ferlay et al., 2018; Sung et al., 2021). Over the past 30 years, the morbidity and mortality of cervical cancer have demonstrated a considerably decreasing tendency in developed countries due to enhanced prevention of cervical cancer and large-scale screening. In contrast, China, as a developing country, has experienced a rising trend in the morbidity and mortality of cervical cancer primarily because of deficiencies in healthcare, especially a neglect of screening (Zeng et al., 2018). Epidemiological evaluations estimated that without intervention, the occurrence of new cervical cancers in China could increase by 40–50% from 2010 to 2050 (Shi et al., 2012). A large body of consistent evidence implicates that persistent infection with high-risk genotypes of human papilloma virus (HR-HPV) is the etiological agent of cervical cancer (Sawaya et al., 2019). Globally, HR-HPV infection is present in 99.7% of patients with cervical cancer (Ngo-Metzger and Adsul, 2019).

HPV infections are common in most sexually active women over their lifetime. Most HPV infections are transient and self-limiting without a clinical approach, whereas a minority of infections can be persis resulting in high-grade precancerous cervical lesions and progressing to cervical cancer (Sawaya et al., 2019). Therefore, early, and regular screening for HPV infection is of great significance for cervical cancer prevention.

As a non-enveloped, double-stranded DNA virus, HPV belongs to the Papilloma genus of the Papovaviridae family, with more than 200 identified genotypes (Dunne and Park, 2013). HPV can be categorised as either HR-HPV or low-risk human papilloma virus (LR-HPV). The top five subtypes of HR-HPV were HPV16, 18, 58, 52, and 33 (Li et al., 2017). Additionally, LR-HPV genotypes are usually associated with proliferative lesions, including genital warts and recurrent respiratory papillomatosis (Sawaya et al., 2019).

Cervical cancer is one of the most preventable and curable cancers worldwide, and vaccination and screening are primary and secondary cervical cancer prevention strategies, respectively. Primary prevention through HPV vaccination is urgently required in several regions of the world. Vaccinating adolescent females and males before their first sexual behaviour is the best way to eliminate HPV infection if HPV vaccination is integrated into national immunisation programs.

More recently, there have been three types of licenced HPV vaccines, including a bivalent vaccine against HPV16 and 18, a quadrivalent vaccine against HPV6, 11, 16, and 18, and a 9-valent vaccine against HPV6, 11, 16, 18, 31, 33, 45, 52, and 58. However, the HPV vaccine cannot prevent all HPV subtypes (Wang et al., 2020). To address this issue, region-specific assessment of HPV subtypes should be implemented to choose an appropriate HPV vaccine to prevent infection more effectively. Cervical cancer screening includes cytological tests and HPV DNA detection. Pathological features observed by cytology can identify cervical disease but cannot absolutely attribute the underlying risk to progression of the identified cervical abnormality or lesion (Gradíssimo and Burk, 2017). In contrast, HPV DNA detection is suitable for identification of the HPV subtype but cannot confirm the cervical abnormality, which has been progressively incorporated into cervical cancer prevention programs based on its noninvasive method and increased sensitivity. It relies on extracting HPV DNA from samples to amplify and identify specific HPV genotypes (Gradíssimo and Burk, 2017). The bivalent human papillomavirus (HPV) 16/18 vaccine was approved by the China Food and Drug Administration in July, 2016 (Pan et al., 2016). Subsequently, quadrivalent vaccine and 9-valent vaccine were successively approved in 2017 and 2018. China introduced the HPV vaccine relatively late and has not yet included it in the national immunization plan. At the same time, due to insufficient supply, the current HPV vaccine coverage rate in China is temporarily lower than the global average (Li et al., 2023).

To effectively guide the use of HPV vaccines and eliminate cervical cancer as much as possible in China, it is essential to investigate the prevalence of HPV and its subtype distribution in specific areas. To date, several studies have demonstrated HPV prevalence in Fujian Province, but the results vary significantly (Wu et al., 2010, 2017). Therefore, we retrospectively investigated the prevalence and genotype distribution of HPV infection in women in Xiamen, Fujian Province. These data provide local epidemiological evidence for immunisation for the prevention of cervical cancer.

## 2. Materials and methods

### 2.1. Study population and sample collection

In this study, Cervical swabs were collected from 47,926 participants aged 16–92 years at the Women and Children’s Hospital, Xiamen University, from November 2019 to June 2021. Two classification methods were used. (1) The participants were divided into six age groups: the G1 group aged <20 years; the G2 group aged ≥20 years but <30 years; the G3 group aged ≥30 years but <40 years; the G4 group aged ≥40 years but <50 years;the G5 group aged ≥50 years but <60 years; and the G6 group aged≥60 years. (2) All patients were divided into two groups: the health examination group (HEG) and gynacological clinic group (GCG). Patients visited the hospital for various reasons, including physical examination, vaginitis, and gynaecological tumours. All women who participated in this study were required to (1) have a sexual history at any age. (2) have not had sexual intercourse or used vaginal drugs in the previous 48 h, and (3) have not been vaccinated against HPV. Women were excluded from this study for having had a diagnosis of cervical cancer, having been pregnancy at the time of enrolment, having had a previous HPV vaccination, lacking age information, having had a hysterectomy, or having immunosuppression. The study was explained to each participant, and written informed consent was obtained. Ethical approval was granted by the Ethics Board of Women and Children’s Hospital, School of Medicine, Xiamen University.

Cervical samples were collected by clinicians using a speculum. A cervical brush was rotated slowly in one direction 6–7 times to obtain sufficient cervical epithelial cells; this was repeated twice. Endocervical and ectocervical cells were collected from the cervical canal using a plastic brush (Hybribio Limited Corp, Chaozhou, Guangdong, China). The brush was placed into a 2 mL vial of Hybribio cervical cell preservation solution (Hybribio Limited Corp, Chaozhou, Guangdong, China) for HPV DNA detection.

### 2.2. DNA extraction, PCR amplification, and HPV genotyping

HPV DNA was extracted by the steps in specification using a Hybribio viral DNA extraction kit (Hybribio Limited Corp, Chaozhou, Guangdong, China). Briefly, the cervical cells were digested with proteinase K. Then, the released DNA was obtained through absorption to magnetic glass particles and washed and purified from these particles using an automated nucleic acid extraction instrument (Hybribio Limited Corp, Chaozhou, Guangdong, China). After DNA extraction, the concentration and purity of the DNA were determined using Nanodrop One (Thermo Fisher Scientific, CA, United States). All samples were stored at 4°C and tested within 48 h. Then, 1 μL of DNA extract was used as a template for PCR amplification. The amplification reagent was configured according to the PCR mixture of 23.25 μL, DNA polymerase 0.75 μL and 1 μL DNA template per person. The following PCR cycling conditions were initiated: 20°C for 10 min and then 95°C for 9 min, followed by 40 cycles of 20 s at 95°C, 30 s at 55°C, 30 s at 72°C. The final extension was performed at 72°C.

A diagnostic kit for human papillomavirus (Hybribio Limited Corp, Chaozhou, Guangdong, China) was used for HPV DNA amplification and genotyping. The kit was used for the qualitative detection of 13 HR-HPV (HPV 16, 18, 31,33, 35, 39, 45, 51, 52, 56, 58,59, and 68) and 18 low-risk HPV (HPV 6, 11, 34, 40, 42, 43, 44, 54, 55, 61, 67, 69, 70, 71, 72, 81, 83, and 84) nucleic acids in women’s cervical exfoliated cells *in vitro*.

The amplified DNA products were then hybridised with biotinylated HPV-genotype-specific probes on nylon membranes. The specific operations were as follows: (1) the hybridisation test reagents were balanced to room temperature, and the hybridisation solution was preheated to 45°C before use. (2) 25 μL of DNA products were heated at 95°C for 5 min, then immediately bathed in ice for 2 min. (3) Added 0.5 mL of the denatured DNA sample solution to the hybrid solution preheated to 45°C. After incubation for 10 min, the pump was turned on for diversion hybridisation. (4) The membrane was washed with 0.8 mL hybrid solution three times at 45°C. (5) 0.5 mL of blocking reagent was added, and the mixture was sealed for 5 min. (6) 0.5 mL of microplate solution was added and incubated for 3.5 min at 25°C. (7) After setting the temperature at 36°C, the nylon membrane was thoroughly washed with 0.8 mL of solution A (mixture of Tris–HCl and 0.1% Tween 20) for four times. (8) The chromogenic solution was added and left to sit for 3–5 min. (9) The nylon membrane was washed with 1 mL of solution B (a mixture of NaCl and 1% SDS) three times, and then rinsed with 2 mL distilled water. (10) The nylon membrane was removed with forceps and placed on absorbent paper, and the genotyping result was analysed by the naked eye within 1 h. A blue dot on the nylon membrane indicated a positive result. Multiple dots showed multiple infections. Based on the arrangement of each genotype of the HPV probe on the nylon membrane, the positive spot was determined as the specific genotype of HPV. We used positive and negative controls provided by the kit for quality control throughout the PCR amplification and hybridisation.

All experimental procedures were performed in accordance with the manufacturer’s instructions.

### 2.3. Statistical analysis

The analyses were performed using SPSS (version 19.0) and WPS (version 2022). Descriptive statistical analyses were performed to determine HPV prevalence and genotype distribution. Single, double, and multiple HPV infections were defined as infections with one, two, or more than two genotypes of HPV. HPV prevalence in designated groups and corresponding 95% confidence intervals (95% CI) were calculated using SPSS 19.0 for Windows (SPSS Inc., IL, USA). For comparisons among different age groups, categorical variables were compared using the chi-square test. The linear-by-linear association test and gamma values were used to assess changes in HR-HPV prevalence across the age groups. Differences were considered statistically significant at *p* values less than 0.05.

## 3. Results

### 3.1. The overall prevalence of HPV infection

From November 2019 to June 2020, 15.13% (7,251/47,926) of participants were tested positive for one or more HPV genotypes. Among the total cases, the HR-HPV infection rate was 11.90% (5,701/47,926), while the LR-HPV infection rate was 3.92% (1,878/47,926). Single HPV infections accounted for 76.83% (5,571/7251) of the positive cases, while double infections accounted for 16.70% (1,211/7251) of the positive cases. Multiple HPV infections accounted for 6.47% (469/7251) of the positive cases.

### 3.2. The prevalence of HPV grouped by age

**Figure 1:**
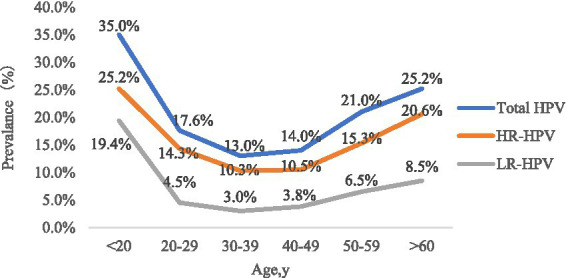
The prevalence of HPV among women in different age groups.

The participants were divided into six groups according to age. The prevalence of HPV infection in each age group was calculated, as shown in Table 1 and Figure 1. The HPV infection rate calculated for each age group ranged from 13.0 to 35.0%. The highest HPV infection rate was observed in G1 (35.0%), followed by G6 (25.2%), G5 (20.1%), G2 (17.6%), G4 (14.0%) and G3 (13.0%). Significant differences in HPV infection rates were found among the age groups (*p* < 0.05). The prevalence initially decreased from the peak observed among women under 20 years old until the 30–39 age group, after which it showed an upward trend from 40–49 age group to >60 age group. Additionally, we observed significant differences among the different age groups in the HR-HPV infection rates for 13 subtypes (HPV16, 18, 31, 33, 35, 39, 45, 51, 52, 56, 58, 59 and 68) (Table 2).

**Table 1:** The prevalence of HPV among women in different age groups.

Group	Age, years	Sample size	Positive no	%(95%CI)
G1	<20	103	36	35.0 (25.7–44.2)
G2	20–29	12,025	2,117	17.6 (16.9–18.3)
G3	30–39	21,842	2,843	13.0 (12.6–13.5)
G4	40–49	9,696	1,359	14.0 (13.3–14.7)
G5	50–59	3,459	694	20.1 (18.7–21.4)
G6	>60	801	202	25.2 (22.2–28.2)

**Table 2:** Infection of patients with 13 subtypes of HR-HPV in different age groups.

HPV subtype	<20 (*n* = 103)	20–29 (*n* = 12,025)	30–39 (*n* = 1,2257)	40–49 (*n* = 5,263)	50–59 (*n* = 1,843)	>60 (*n* = 398)	*χ* ^2^	*p*	Gamma value
	36	2,117	2,843	1,359	694	202	536.037	<0.001	0.218
HPV-16	7 (6.8%)	215 (1.8%)	221 (1.8%)	77 (5.7%)	49 (2.7%)	22 (5.5%)	4.955	0.026	0.032
HPV-18	6 (5.8%)	116 (1.0%)	142 (1.2%)	63 (4.6%)	19 (1.0%)	8 (2.0%)	1.301	0.003	-0.139
HPV-31	4 (3.9%)	59 (0.5%)	56 (0.5%)	26 (1.9%)	14 (0.8%)	6 (1.5%)	1.701	0.192	0.040
HPV-33	4 (3.9%)	94 (0.8%)	108 (0.9%)	49 (3.6%)	23 (1.2%)	12 (3.0%)	8.961	0.003	0.103
HPV-35	0 (0%)	20 (0.2%)	36 (0.3%)	16 (1.2%)	12 (0.7%)	4 (1.0%)	18.170	<0.001	0.301
HPV-39	0 (0%)	163 (1.4%)	166 (1.4%)	81 (6.0%)	54 (2.9%)	5 (1.3%)	12.443	<0.001	0.106
HPV-45	0 (0%)	55 (0.5%)	69 (0.6%)	21 (1.5%)	20 (1.1%)	3 (0.8%)	4.523	0.033	0.104
HPV-51	5 (4.9%)	182 (1.5%)	165 (1.3%)	85 (6.3%)	55 (3.0%)	10 (2.5%)	9.819	0.002	0.075
HPV-52	9 (8.7%)	399 (3.3%)	497 (4.1%)	221 (16.3%)	119 (6.5%)	43 (10.8%)	58.983	<0.001	0.141
HPV-56	1 (1.0%)	87 (0.7%)	150 (1.2%)	58 (4.3%)	38 (2.1%)	18 (4.5%)	47.847	<0.001	0.243
HPV-58	5 (4.9%)	266 (2.2%)	301 (2.5%)	121 (8.9%)	62 (3.4%)	29 (7.3%)	16.846	<0.001	0.083
HPV-59	2 (1.9%)	92 (0.8%)	120 (1.0%)	53 (3.9%)	36 (2.0%)	10 (2.5%)	22.800	<0.001	0.175
HPV-68	1 (1.0%)	95 (0.8%)	111 (0.9%)	55 (4.0%)	26 (1.4%)	8 (2.0%)	10.866	0.001	0.129

### 3.3. Overall and distribution of single, double, and multiple HPV infections

In this study, single HPV infection was the most frequently pattern, accounting for 76.83% of cases. Double (16.70%) and multiple (6.47%) HPV infections were relatively rare. According to our classification by age (Table 3), the highest single HPV infection rate was in G1 (21.4%), followed by G6 (17.5%), G5 (15.3%), G2 (12.9%), G4 (12.0%), and G3 (10.9%). For double infections, the highest HPV infection rate was observed in G6 (4.5%) and the lowest in G4 (1.6%). The two genotypes frequently found in double infection were HPV52 + HPV58. Consistent with single infection, multiple infections had the highest infection rate in G1 (9.7%). The highest multiple infection in our study was 10 genotypes in the same patient.

**Table 3:** HPV prevalence according to age groups.

HPV infection	Age group, years						Total (*n* = 47926)	*χ* ^2^	*p* value
	<20 (*n* = 103)	20–29 (*n* = 12,025)	30–39 (*n* = 21,842)	40–49 (*n* = 9,696)	50–59 (*n* = 3,459)	>60 (*n* = 801)			
HPV	36 (35.0%)	2,117 (17.6%)	2,843 (13.0%)	1,359 (14.0%)	694 (20.1%)	202 (25.2%)	7,251	303.312	<0.001
Single HPV	22 (21.4%)	1,557 (12.9%)	2,374 (10.9%)	1,163 (12.0%)	530 (15.3%)	140 (17.5%)	5,786	103.375	<0.001
Double HPV	4 (3.9%)	400 (3.3%)	374 (1.7%)	155 (1.6%)	110 (3.2%)	36 (4.5%)	1,079	143.881	<0.001
Multiple HPV	10 (9.7%)	160 (1.3%)	95 (0.4%)	41 (0.4%)	54 (1.6%)	26 (3.2%)	386	283.435	<0.001

### 3.4. HPV genotype distribution

**Figure 2:**
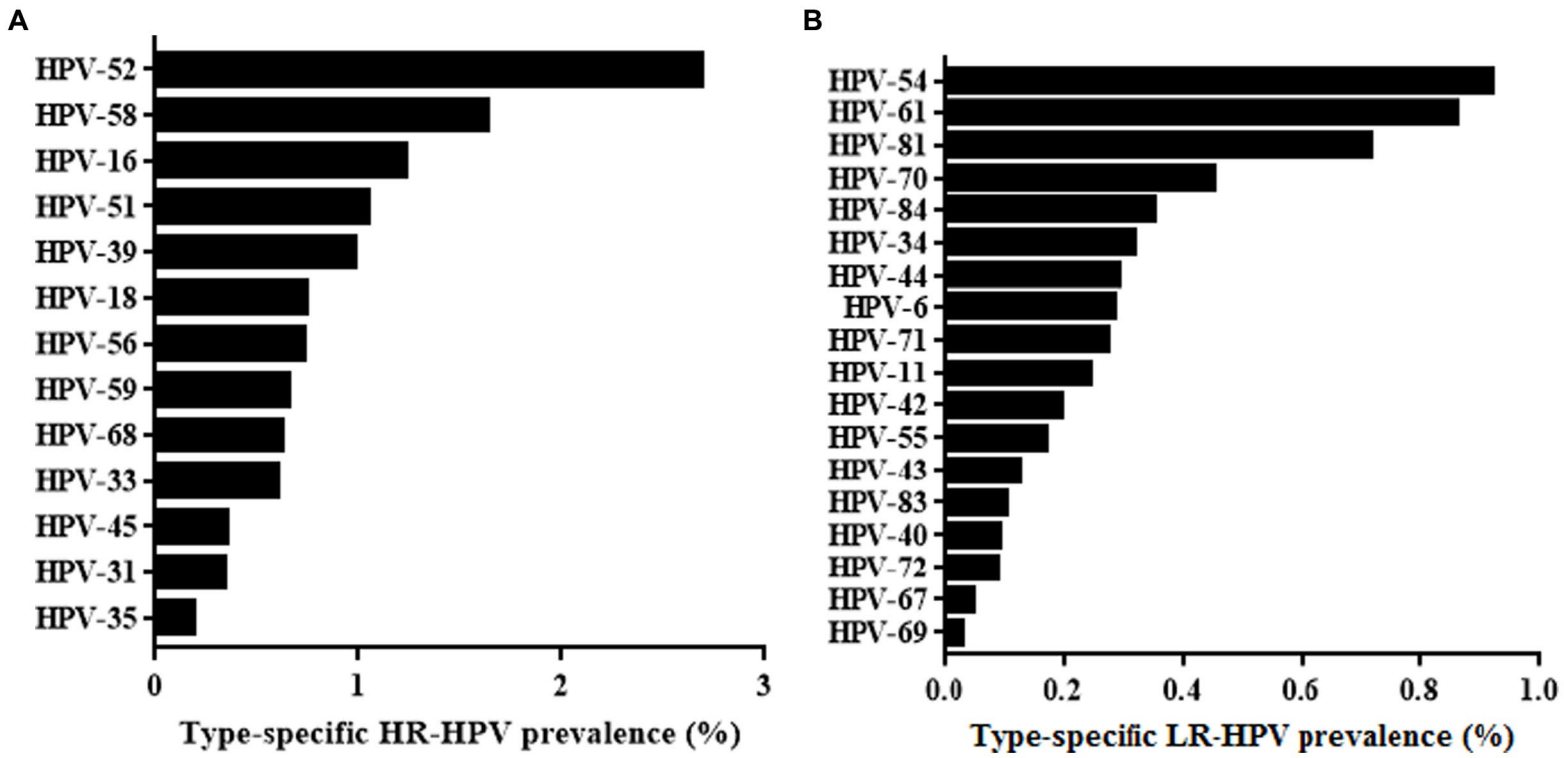
Overall infection rate of different HR-HPV **(A)** and LR-HPV **(B)** genotypes.

Thirteen HR-HPV and 19 LR-HPV subtypes were included in this study. Among the HPV-positive cases shown in Figure 2A, the following HR-HPV genotypes were most common: HPV52(2.69%), HPV58 (1.63%), HPV16 (1.23%), HPV51 (1.05%) and HPV39 (0.98%). It is worth noting that HPV18 only ranked seventh in HR-HPV cases, which is different from the results of previous studies (Wang et al., 2018). For LR-HPV, HPV-54 (0.92%), HPV61 (0.86%), HPV81 (0.71%), and HPV70 (0.45%) were most common (Figure 2B).

For participants with single HPV infection, the top five HR-HPV genotypes were HPV52, HPV58, HPV16, and HPV39. For double HPV infections, the most common HR-HPV was HPV52, followed by HPV58, HPV16, and HPV 51. For individuals with multiple HPV infections, the HR-HPV that ranked among the top five were HPV52, HPV58, HPV51, HPV16, and HPV39 (Table 4).

**Table 4:** Distribution of HR-HPV genotypes in study participants.

HPV subtype	Single infection		Double infections		Multiple infections		Total infections
	Positive no	% (95% CI)	Positive no	% (95% CI)	Positive no	% (95% CI)	% (95% CI)
HPV-52	380	0.79 (0.71–0.87)	279	0.58 (0.51–0.65)	158	0.33 (0.28–0.38)	1.70 (1.59–1.82)
HPV-58	183	0.38 (0.33–0.44)	201	0.42 (0.36–0.48)	93	0.19 (0.15–0.23)	1.00 (0.91–1.08)
HPV-16	151	0.32 (0.26–0.37)	141	0.29 (0.26–0.34)	77	0.16 (0.12–0.20)	0.77 (0.69–0.85)
HPV-39	109	0.23 (0.18–0.27)	119	0.25 (0.20–0.29)	58	0.12 (0.09–0.15)	0.60 (0.53–0.57)
HPV-51	58	0.12 (0.09–0.15)	128	0.27 (0.20–0.29)	88	0.18 (0.15–0.22)	0.57 (0.50–0.64)
HPV-18	95	0.20 (0.16–0.24)	71	0.15 (0.11–0.18)	45	0.09 (0.07–0.12)	0.44 (0.38–0.50)
HPV-56	63	0.13 (0.10–0.16)	73	0.15 (0.08–0.14)	53	0.11 (0.08–0.14)	0.39 (0.34–0.45)
HPV-59	87	0.18 (0.14–0.22)	54	0.11 (0.08–0.14)	39	0.08 (0.06–0.11)	0.38 (0.32–0.43)
HPV-68	79	0.16 (0.13-0.20)	64	0.13 (0.10–0.17)	38	0.08 (0.05–0.10)	0.38 (0.32–0.43)
HPV-33	47	0.10 (0.07–0.13)	72	0.15 (0.12–0.18)	45	0.09 (0.07–0.12)	0.34 (0.29–0.39)
HPV-45	58	0.12 (0.09-0.15)	26	0.05 (0.03–0.08)	16	0.03 (0.02–0.05)	0.21 (0.17–0.25)
HPV-31	12	0.03 (0.01–0.04)	42	0.09 (0.06–0.11)	34	0.07 (0.05–0.09)	0.18 (0.15–0.22)
HPV-35	23	0.05 (0.03–0.07)	14	0.03 (0.01–0.04)	12	0.03 (0.01–0.04)	0.10 (0.07–0.13)

### 3.5. Prevalence and distribution of HPV infection in HEG and GCG

**Figure 3:**
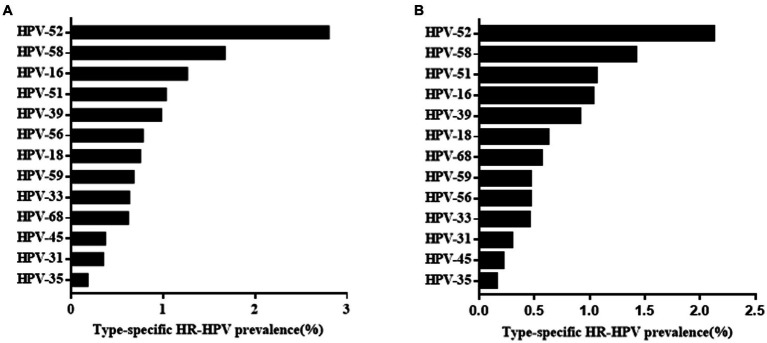
Overall infection rate of different HR-HPV genotypes in GCG group **(A)** and HEG group **(B)**.

**Figure 4:**
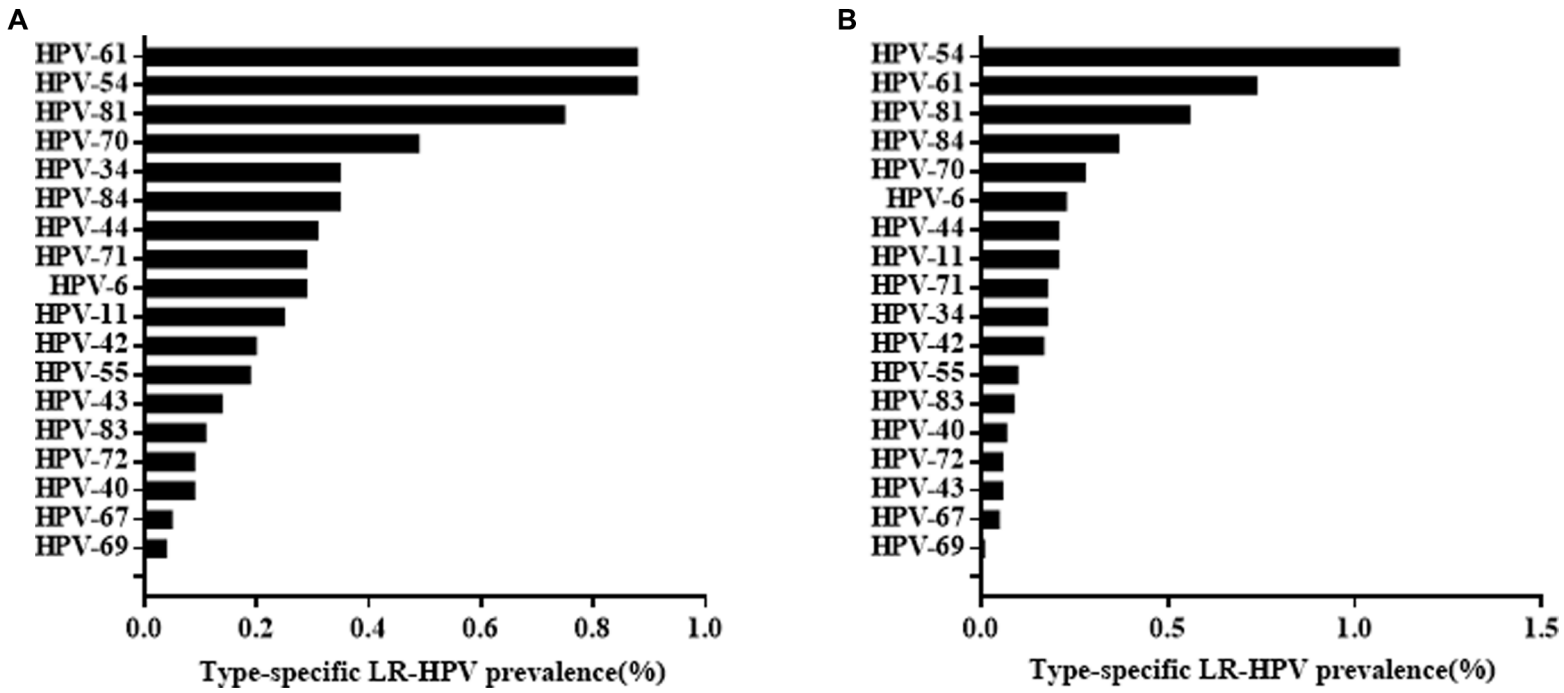
Overall infection rate of different LR-HPV genotypes in GCG group **(A)** and HEG group **(B)**.

Among the 47,926 HPV screening tests, the specimens were divided into the HEG (n = 39,713) and GCG (n = 8,213). The HPV infection rate in the GCG was 15.72%, whereas that in the HEG was 12.29%. As shown in Table 5, the HPV infection rate in the GCG was significantly higher than that in the HEG (*p* < 0.001). The distribution of HR-HPV subtype infection rates for GCG and HEG was shown in Figure 3. The top five HR-HPV subtypes in the GCG group were HPV52, HPV58, HPV16, HPV51, and HPV39. For the HEG, the most common HR-HPV subtypes were HPV52, HPV 58, HPV51, HPV16, and HPV39. The top three LR-HPV types in the GCG and HEG were HPV54, HPV61, and HPV81 (Figure 4).

**Table 5:** The prevalence of HPV among women in different groups.

		HPV(−)	HPV(+)	Total	*χ* ^2^	*p* value
Group	GCG	33,471	6,242	39,713	62.441	<0.001
	HEG	7,204	1,009	8,213		
Total		40,675	7,251	49,726		

## 4. Discussion

In November 2020, the World Health Organization (WHO) launched a strategy to reduce the current worldwide incidence of HPV from 13.3 per 100,000 (age-adjusted) women to 4 per 100,000 women by 2030 as the first step towards the elimination of cervical cancer (World Health Organization, 2020). Recently, the National Health Commission of China published a cervical cancer screening program regarding the introduction of primary HPV testing and cytology tests, the focus of which is on rural and urban subsistence allowance women (Zhang et al., 2021). By the end of 2025, more than half of women of an appropriate age will be screened for cervical cancer. This program will promote the early diagnosis and treatment of cervical cancer. In China, the prevalence of the disease is still relatively high due to inadequate vaccination and HPV screening coverage, especially in rural areas (Ma et al., 2020). In addition, the prevalence and genotype distribution of HPV differs by geographic area (Li et al., 2019). There are few reports on the prevalence of HPV in Fujian Province, especially in Xiamen. As an inflowing city, the population composition of Xiamen is progressively complex because of its numerous migrant populations. Thus, it is of great significance to understand the overall HPV prevalence and genotype distribution to formulate prevention efforts and strategies for the elimination of cervical cancer.

Among the participants in this study, the overall HPV positive rate, including HR-HPV and LR-HPV, was 15.13%, which is consistent with a study demonstrating the overall prevalence of HPV infection in China (15.54%) (Zhu et al., 2019). Some studies from other cities in China have also reported similar HPV prevalence (Wu et al., 2013; Hong et al., 2015; Han et al., 2021; Li et al., 2021; Yu et al., 2021). However, the HPV prevalence reported was higher than that in Shannan (8.16%) (Feng et al., 2022) and Xinjiang (9.34%) (Yan et al., 2020), but lower than that in Zhejiang Province (22.3%) (Yan et al., 2021), Beijing (22.7%) (Zhu et al., 2021), Jiangxi Province (22.49%) (Zhong et al., 2017), and Heilongjiang Province (27.1%) (Liu et al., 2020). The variability in HPV prevalence may be accounted for by diverse geographical conditions and economic developmental levels. Compared to the two studies that reported results in Fujian Province, we found that our results were significantly lower than those of Fuzhou (38.3%) (Wu et al., 2017) and Quanzhou (22.5%) (Wu et al., 2010). This suggests that the prevalence of HPV in Fujian Province decreased significantly from 2009 to 2021. Considering that the same laboratory method (flow-through hybridisation technique) used in these reports, discrepancies in the results might be attributed to differences in the study period and the progressive clinical use of HPV vaccination.

Age-specific HPV infections are pivotal for the next stage of cervical cancer prevention. Numerous reports have shown that the HPV infection rate is significantly age-specific. In the present study, we found that women under 20 years of age had the highest HPV infection rate. Women in the >60 years age group had a smaller peak of HPV infection rate peak. HPV infection in women aged 20–49 years gradually decreased, reached the lowest in the age group of women who were 30–39 years old, and then showed a progressively upward trend. In conclusion, age-specific HPV distribution in our study was shown as a bimodal “U” curve, in line with most other reports (Li et al., 2021; Zhu et al., 2021). There are possible reasons for this phenomenon. On the one hand, young women may have been sensitive to HPV due to relatively frequent sexual activity without protective measures and their non-sensitised immune systems (Wissing et al., 2019). However, it has been reported that the majority of young women infected with HPV are transient, and that their immune systems protect them from persistent HPV infection (Gravitt and Winer, 2017). Hence, the HPV infection rate gradually declines with age. On the other hand, for women aged over 60 years, continued infection with the virus or latent HPV reactivation due to physiological disorders, such as hormonal level changes, may result in immune disorders, presenting as a risk factor for cervical cancer development (Malagón et al., 2018).

Furthermore, we observed that women aged under 20 years old and aged over 60 years old were more sensitive to HPV, whether for single, double, or multiple infection. Host susceptibility and virus characteristics may account for this phenomenon (Stanley, 2010). Therefore, it is pivotal for adolescents to be vaccinated to reduce HPV primary infection. In addition, it is recommended that aged women should involve in cervical cancer screening regularly.

In the present study, we analysed the age-specific distribution of single, double, and multiple HPV types. Whether multiple infections increase the risk of cervical cancer is yet to be determined. Some studies demonstrated that multiple infections displayed a longer duration of HPV infection compared to a single infection, which may result in cervical cancer occurrence (Kim et al., 2021). On the contrary, some studies found that single HPV infection had a higher risk of developing into cervical cancer than multiple infections, the possible pathogenic mechanism of which could be competition or a counterbalance between various HPV subtypes (Nie et al., 2016; Li et al., 2019). In our study, the prevalence of single HPV infections was higher than that of double and multiple HPV infections. More attention should be paid to HPV52 infection because of its high proportion of single, double, and multiple HPV infections. The investigation of double and multiple HPV infections is conductive to providing a closer view of HPV prevalence. Furthermore, the investigation of double and multiple HPV infections is important to guide the development of a second-generation multivalent HPV vaccine in the future. The specific mechanism by which single or multiple HPV infections increase the risk of developing cervical cancer warrants further investigation.

The most prevalent HPV genotypes among women vary significantly in different regions. Therefore, specific HPV prevalence data are closely related to future vaccine developments. According to an HPV epidemiological report that was carried out in mainland China, the five most common HPV subtypes were HPV16, 52, 58, and 18 (Li et al., 2019). Other studies have reported high HPV infection rates of HPV16, HPV52, and HPV58 in northern Henan (Wang et al., 2022). However, similar to the study concerning Jilin (Hao et al., 2020), we found a HPV genotype distribution in which HPV52 was the most prevalent in this area, followed by HPV58, 16, and 51. Seventy percent of the HR-HPV DNA detected in patients with cervical cancer was HPV16 and 18. HPV16 was ranked third in our study. Notably, HPV18 only ranked seventh in HR-HPV to be detected, which is consistent with recent studies that reported data in other regions (Jiang et al., 2019; Yang et al., 2020; Wang et al., 2022). This phenomenon could be due to the bivalent vaccine against HPV16 and 18 applied in clinical practice. In addition to HPV16 and 18, both HPV52 and 58 have been reported to be potent oncogenic factors that induce intraepithelial neoplasia (Wang et al., 2018; Li et al., 2019). In addition to HR-HPV, the present study investigated the prevalence of LR-HPV. We found that HPV54, 61, and 81 were the most common LR-HPV types, none of which were targeted by the present vaccines. Unlike HR-HPV, LR-HPV has few associations with cervical cancer but results in proliferative lesions (Ferlay et al., 2018). HPV vaccination is not only conducive to women’s health but can also reduce medical expenses. The present study supports a recommendation for use of the 9-valent vaccine HPV vaccine in Xiamen. Meanwhile, vaccines targeting HPV51 and 39 should be the subject of future research to efficiently protect against HR-HPV infection.

Since the participants in this study included outpatients and health examination subjects, we analysed the total HPV positivity rate and distribution of HPV subtype infection rate in the two different groups. We found that HPV infection rate was higher in outpatients than in health examination subjects. Both groups showed similar HPV subtype distributions. This result of this study was in line with previous literature which means that there was no obvious HPV subtype characteristic when one considered whether to attend gynaecology clinics (Yan et al., 2021).

Although this study reported large-scale information on HPV prevalence and genotype distribution in Xiamen, it has some limitations that should be addressed. First, our results were not combined with cytology results. Cervical or histological results could not be obtained for the women who were included in this study. Therefore, we could not associate HPV infection with the genotype distribution of different cervical abnormalities. Second, the personal information of the patients was not recorded in this study, so that we couldn not specify the impact of different backgrounds on the rate of HPV infection and genotype distribution. Third, the design of our study was cross-sectional, and a temporal trend of HPV prevalence is recommended to provide better conditions for further research.

## 5. Conclusion

In summary, HPV52, 58, 16, and 51 were the predominant HR-HPV subtypes in Xiamen. These findings provide fundamental information for cervical cancer screening and valuable guidance for local governments to promote next-generation HPV-targeted vaccination in the future.

## Data availability statement

The original contributions presented in the study are included in the article/supplementary material, further inquiries can be directed to the corresponding author.

## Ethics statement

The studies involving human participants were reviewed and approved by Women and Children’s Hospital Affiliated to Xiamen University. Written informed consent to participate in this study was provided by the participants and minors’ legal guardian/next of kin.

## Author contributions

YS, YH, and YG designed and supervised the research. YS, WW, LZ, and XH acquisition of data. YS, JZ, and XC analyzed and interpreted the data. YS prepared the manuscript. All authors contributed to the article and approved the submitted version.

## Funding

This study was supported by the Medical and Health guiding project of Xiamen (grant no. 3502Z20224ZD1219) and National Natural Science Foundation of China (grant no. 82101955).

## Conflict of interest

The authors declare that the research was conducted in the absence of any commercial or financial relationships that could be construed as a potential conflict of interest.

## Publisher’s note

All claims expressed in this article are solely those of the authors and do not necessarily represent those of their affiliated organizations, or those of the publisher, the editors and the reviewers. Any product that may be evaluated in this article, or claim that may be made by its manufacturer, is not guaranteed or endorsed by the publisher.
